# Discovery of Novel c-Jun N-Terminal Kinase 1 Inhibitors from Natural Products: Integrating Artificial Intelligence with Structure-Based Virtual Screening and Biological Evaluation

**DOI:** 10.3390/molecules27196249

**Published:** 2022-09-22

**Authors:** Ruoqi Yang, Guiping Zhao, Bin Yan

**Affiliations:** 1 College of Pharmacy, Shandong University of Traditional Chinese Medicine, Jinan 250355, China; 2 Institute of Chinese Materia Medica, China Academy of Chinese Medical Sciences, Beijing 100700, China

**Keywords:** JNK1, natural products, virtual screening, artificial intelligence, computer-aided drug design

## Abstract

c-Jun N-terminal kinase 1 (JNK1) is currently considered a critical therapeutic target for type-2 diabetes. In recent years, there has been a great interest in naturopathic molecules, and the discovery of active ingredients from natural products for specific targets has received increasing attention. Based on the above background, this research aims to combine emerging Artificial Intelligence technologies with traditional Computer-Aided Drug Design methods to find natural products with JNK1 inhibitory activity. First, we constructed three machine learning models (Support Vector Machine, Random Forest, and Artificial Neural Network) and performed model fusion based on Voting and Stacking strategies. The integrated models with better performance (AUC of 0.906 and 0.908, respectively) were then employed for the virtual screening of 4112 natural products in the ZINC database. After further drug-likeness filtering, we calculated the binding free energy of 22 screened compounds using molecular docking and performed a consensus analysis of the two methodologies. Subsequently, we identified the three most promising candidates (Lariciresinol, Tricin, and 4′-Demethylepipodophyllotoxin) according to the obtained probability values and relevant reports, while their binding characteristics were preliminarily explored by molecular dynamics simulations. Finally, we performed in vitro biological validation of these three compounds, and the results showed that Tricin exhibited an acceptable inhibitory activity against JNK1 (IC_50_ = 17.68 μM). This natural product can be used as a template molecule for the design of novel JNK1 inhibitors.

## 1. Introduction

The c-Jun N-terminal kinases (JNKs), members of the mitogen-activated protein kinase (MAPK) family, can be activated by upstream MKK4/7 kinases and are widely involved in many biological processes such as cell proliferation and apoptosis [1]. Studies have shown that JNKs are encoded by three genes, of which JNK1 and JNK2 are expressed in a variety of organs, whereas JNK3 is mainly expressed in the brain [2,3]. As one of the three isoforms of the JNKs, the structure of JNK1 is similar to other closely related kinases and contains three main parts: an N-terminal lobe with β strands, a C-terminal lobe with α helices, and a hinge region connecting them [4]. The over-expression of JNK1 is considered a key mediator of metabolic disorders, and there have been many reports about this target in the treatment of type-2 diabetes [5]. However, the development of JNK1 inhibitors is still in its infancy. Most of the common JNK1 inhibitors are type I inhibitors, and they mainly block the phosphorylation process of the kinase by occupying the highly conserved ATP-binding pocket and forming hydrogen bonds with the key residue MET-111. The search for novel inhibitors targeting JNK1 is a valuable research direction.

The high diversity of natural products in terms of chemical structures and physicochemical properties makes them a precious source for the discovery of novel active chemicals [6]. Compared to synthetic compounds, the structures of natural products are more complex. It is also due to their unique structural characteristics that many natural products are used as a starting point for drug design [7]. In the pharmaceutical industry, high-throughput screening (HTS) and virtual screening (VS) are two tools frequently adopted for the identification of lead compounds. Experiment-based HTS is known to be an inefficient and lengthy process. In contrast, computation-based VS allows for the rapid screening of large compound databases with less time and money investment [8]. However, there are some problems that cannot be ignored. For example, molecular docking, a representative technique of VS, has a generally high rate of false positive results. In recent years, artificial intelligence (AI) techniques, including machine learning and deep learning, have become increasingly sophisticated in the search for novel active compounds. Studies have shown that combining emerging AI with traditional VS can overcome the shortcomings of individual methods, such as high false positive rates or slow computational speed, thus assisting computational chemists to select lead compounds more reliably [9]. Therefore, the integration of multiple virtual screening strategies facilitates the balance of efficiency and accuracy.

As over-expression of kinases is a major trigger for the development of many diseases, the search for inhibitors targeting different kinases has become one of the hotspots in the field of drug discovery. Zhu et al. performed a virtual screening of glycogen synthase kinase-3 beta (GSK3β) in a parallel manner, combining multiple computational tools including molecular docking, pharmacophore modeling, and naive Bayesian classification. Two potential inhibitors were eventually identified through an in vitro kinase assay [10]. Che et al. significantly improved the performance of molecular docking and pharmacophore-based virtual screening using the AI method, which not only reduced the number of false positive compounds but also successfully identified four active interleukin-1 receptor associated kinase-1 (IRAK1) inhibitors [11]. However, to our knowledge, there is no relevant report on the discovery of natural products targeting JNK1 by integrating computational strategies. In this study, we constructed a sequential “multi-stage virtual screening” system. First, we rapidly identified potential JNK1 inhibitors with desirable pharmacokinetic profiles from thousands of natural products based on machine learning classification models and ADMET prediction. Then, we used structure-based molecular docking to calculate the corresponding binding free energies. After the results of two rounds of screening were analyzed by consensus, the candidate compounds with high scores were selected. Subsequently, the action mechanism and binding stability of these inhibitors were further explored by binding mode analysis and molecular dynamics simulation. Finally, the reliability of the protocol was verified by biological assay.

## 2. Results

### 2.1. Chemical Space Distribution

**Figure 1:**
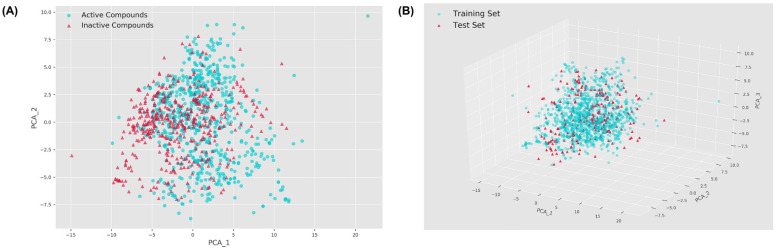
Visualization of the two-dimensional distribution of active and inactive compounds (**A**) and the three-dimensional distribution of training and test sets (**B**).

In general, using a dataset with a broad chemical space to build a model, as well as a reasonable division of the training and test sets, can facilitate the model in better learning the molecular characteristics of active compounds and, thus, improve the prediction performance. In the present study, we visualized the dataset by a principal component analysis (PCA) algorithm in two-dimensional and three-dimensional spaces, where the former was used to analyze the distribution of active and inactive compounds, and the latter was used to analyze the partitioning of the training and test sets [12]. After the feature selection step, a total of 120 descriptors were finally used for PCA. Their specific information is listed in Table S1. From Figure 1A, we can see that the spatial regions of active and inactive compounds in the JNK1 dataset are mostly overlapping, mainly distributed in the range of −10 to 10 and −7.5 to 7.5 for PCA_1 and PCA_2, respectively. In addition, we also notice that the compounds in the dataset are diverse in molecular structure, which implies that the application domain of the model will be relatively wide. As shown in Figure 1B, the chemical spaces of the training and test sets are very similar, mainly distributed between −10 and 5 for PCA_1, −2.5 and 7.5 for PCA_2, and −7.5 and 2.5 for PCA_3. These results indicate that the division method is feasible.

### 2.2. Establishment and Comparison of Machine Learning Models

**Figure 2:**
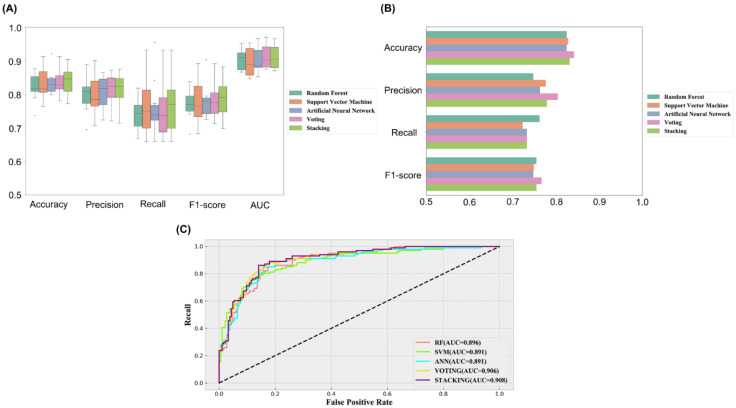
(**A**) The five models’ prediction performance in 10-fold cross-validation for training set. (**B**,**C**) The five models’ prediction performance for test set.

Three machine learning models were constructed using the molecular descriptors after feature selection, and the hyperparameters were tuned on the training set by the Bayesian optimization algorithm combined with 10-fold cross-validation. The combination of hyperparameters for each model was set as follows: n_estimators = 283, max_depth = 25, and max_features = 0.231 in the RF model; kernel = “rbf”, C = 4.691, and gamma = 0.013 in the SVM model; hidden_layer_sizes = 201 and alpha = 0.155 in the ANN model. The prediction performance of each model on the training set is shown in Figure 2A, where we can see that the average accuracy of all models was higher than 82%. Generally, lower precision reflects a higher rate of false positives and lower recall represents a weaker ability of the model to discriminate positive samples. In this study, we focused more on the precision of the model, considering that our aim was to increase the proportion of true positive samples among the screened compounds as much as possible. In addition, among the metrics of machine learning models, AUC is more informative in that it provides a reasonable evaluation of the performance of the classifier, and the closer the AUC value of a model is to 1, the stronger its predictive power. In the three models generated for JNK1, the average precision was greater than 79% and the average AUC values exceeded 0.89. The generalization ability reflects the prediction performance of the machine learning models on the unknown dataset, which is usually more valuable than that on the training set. As shown in Figure 2B,C, the performance of the three models on the test set was comparable to that on the training set, which indicates that the models were not overfitted and the prediction results were relatively reliable.

As each algorithm predicts active compounds based on distinct principles and there is no significant difference in the prediction performance of the constructed individual models, we next integrated the three classifiers using two model fusion strategies. Voting is a simple and effective fusion method that can weight the prediction results of individual models to ensure performance while improving model diversity. Stacking is a more powerful learning-based fusion strategy that uses the initial dataset to train the “component learner” and then generates a new dataset for training the “meta-learner”. The results are shown in Table 1; the integrated models generally outperformed the single models in terms of prediction performance on the training and test sets, with AUC values of 0.906 and 0.908, as well as overall accuracies of 84.2% and 83.2%, respectively. This phenomenon demonstrates that the integrated models based on Voting and Stacking strategies have more desirable discrimination abilities and are more suitable for virtual screening of small-molecule databases.

### 2.3. Feature Importance Ranking

To better explain the potential association between each molecular descriptor and compound activity, we obtained the 10 descriptors with the highest correlation based on the ranking of feature importance in the RF model. For the JNK1 activity prediction model, SlogP_VSA8, BCUT2D_MWLOW, and Kappa3 were the features with relatively high importance, which also indicated that they contributed more to the prediction of the machine learning model (Figure S1). These descriptors could provide important guidance for the discovery of potential inhibitors.

### 2.4. Integrated Model-Based Activity Prediction and Drug-Likeness Filter

Following the generation of machine learning models and evaluation of their predictive performance, the activity prediction of 4112 named natural products in the ZINC database was performed using the integrated models. The 208 RDKit descriptors were first calculated for all molecules, and then the same data preprocessing and feature selection steps were performed. To further improve the reliability of the screening results, we set the threshold of both integrated models to 0.8, which finally showed a total of 343 compounds predicted as JNK1 inhibitors. It should be noted that the results will be useful only if the predicted molecules are located in the chemical space of the trained molecules. Therefore, we applied PCA on the 4112 predicted natural products and saw whether they lay within the range of the training set. The results showed that the chemical space distribution of most of the predicted compounds overlapped with that of the trained compounds, which also implies that the virtual screening results based on machine learning are reliable (Figure S2).

**Figure 3:**
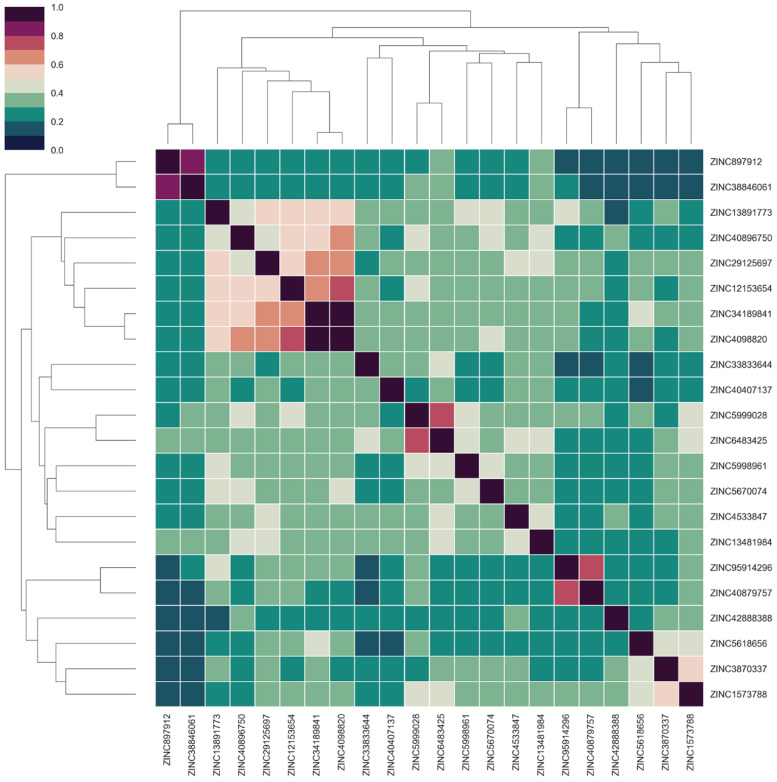
Correlation clustering heatmap for screening compounds.

Subsequently, we predicted the ADMET properties of these compounds and further filtered them according to the set criterion. Specifically, the desirable compounds should satisfy the Lipinski’s Rule of Five and have the value of Log of the aqueous solubility (LogS) between −4 and 0.5, the value of Human Intestinal Absorption (HIA) greater than 30%, the value of Medicinal Chemistry Evolution (MCE-18) greater than 45, and the ability to penetrate the Blood–Brain Barrier (BBB). In addition, all compounds should have no risk of toxicity, including mutagenicity, carcinogenicity, hepatotoxicity, and oral acute toxicity. We finally obtained 22 drug-like molecules with acceptable ADMET properties, and the correlation clustering analysis among them is shown in Figure 3. The overall similarity among the proposed hits was low, and the matrix color was relatively green (less than 0.5), while the results of hierarchical clustering indicated that these compounds showed diversity in chemical structure.

### 2.5. Virtual Screening Based on Molecular Docking

**Figure 4:**
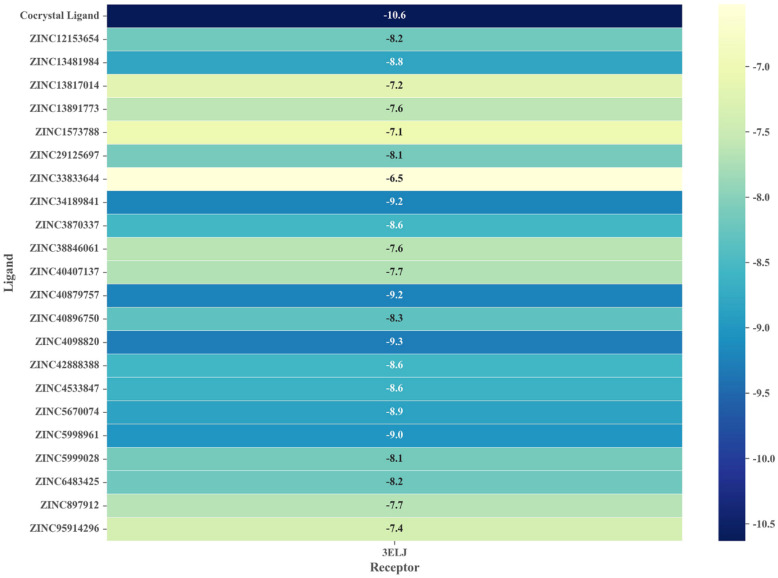
The affinity of 22 screened hits to the target protein (PDB ID: 3ELJ).

Molecular docking is one of the most commonly used techniques in computer-aided drug design (CADD) to predict the binding mode of small molecules in the active pocket of target proteins and to calculate the affinity between them. To ensure the reliability of the docking protocol adopted (i.e., whether the true binding conformation between ligand and receptor can be correctly predicted), we performed re-docking and calculated the RMSD between the docking pose and the co-crystallized conformation [13]. In general, if the RMSD value is ≤2.0 Å, it means that the prediction performance of molecular docking is satisfied. In the present study, the RMSD value of the re-docking was 1.27 Å, which indicates that the docking process is reliable. We then docked the candidate compounds obtained after the first round of screening into the binding site of the target protein, and the binding free energies are shown in Figure 4. Specifically, 14 out of 22 candidate compounds had binding free energies below −8.0 kcal/mol, among which ZINC4098820 exhibited the highest affinity (−9.3 kcal/mol). It is worth noting that the affinities of these hits were lower than those of the co-crystallized ligand (−10.6 kcal/mol), which suggests that their efficacy could be improved by further molecular modification.

### 2.6. Identification of Candidate Compounds

Reducing the proportion of inactive compounds in the virtual screening results not only enhances the reliability of the whole process but also saves expenses and time for the subsequent biological evaluation. In this study, we obtained the rankings of 22 candidate compounds for the consensus analysis based on self-defined criteria, and 13 of them have a total score greater than 0.8, which means they have the potential to become lead compounds for JNK1 inhibitors. Details of the top five compounds are shown in Table 2. By searching the relevant literature, we found relatively few reports on Nigracin (ZINC40879757) and Ophiopogonanone E (ZINC13481984). Considering the feasibility and application value, we focused on three compounds: Lariciresinol (ZINC4098820), Tricin (ZINC5998961), and 4′-Demethylepipodophyllotoxin (ZINC5670074), which have excellent effects in anti-inflammation, anti-tumor, and anti-infection [14,15,16].

### 2.7. Analysis of Drug–Protein Interaction

**Figure 5:**
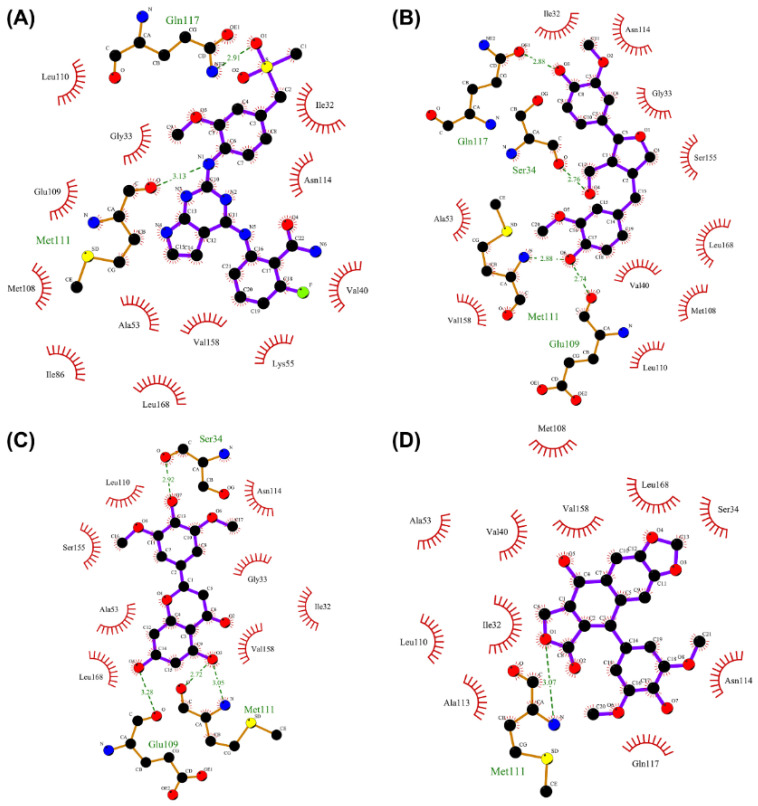
Two-dimensional presentations of the interactions between ligands and JNK1. (**A**) Co-crystallized ligand, (**B**) Lariciresinol, (**C**) Tricin, and (**D**) 4′-Demethylepipodophyllotoxin.

**Figure 6:**
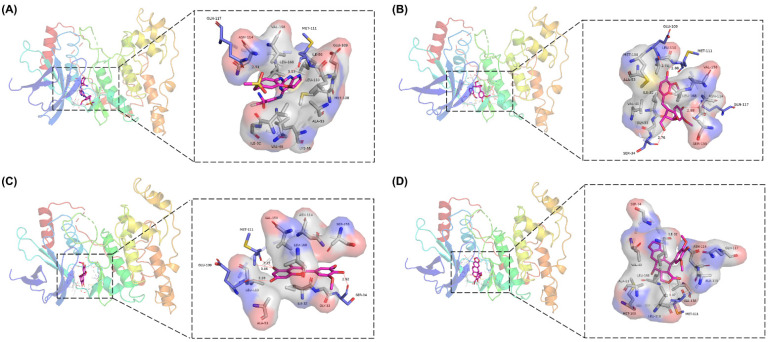
Three-dimensional presentations of the interactions between ligands and JNK1. (**A**) Co-crystallized ligand, (**B**) Lariciresinol, (**C**) Tricin, and (**D**) 4′-Demethylepipodophyllotoxin.

To further elucidate the binding modes of the candidate inhibitors to the target protein, we analyzed the interactions of these three compounds with key amino acids using LigPlot^+^ software, and the 2D/3D views are illustrated in Figure 5 and Figure 6, respectively. Previous studies have shown that the hydrogen bonding interaction formed by the small-molecule ligand with the hinge region residue MET-111 may be crucial for the inhibitory activity against JNK1 [17]. Lariciresinol exhibited the strongest binding affinity to JNK1, and the hydroxyl groups on its two sides of the benzene ring established hydrogen bonding interactions with three residues GLU-109, MET-111, and GLN-117, while another hydrogen bond was observed between residue SER-34 and the hydroxyl group on the tetrahydrofuran (Figure 5A and Figure 6A). Likewise, the two hydroxyl groups on the benzene ring of Tricin formed three hydrogen bonds with two residues GLU-109 and MET-111, while one hydroxyl group on the other side of the benzene ring interacted with residue SER-34 through another hydrogen bond (Figure 5B and Figure 6B). 4′-Demethylepipodophyllotoxin also formed one hydrogen bond with residue MET-111 (Figure 5C and Figure 6C). In addition, several residues including ILE-32, VAL-158, LEU-168, and ALA-53 were involved in the formation of hydrophobic interactions. These residues are essential for the stable binding of drugs and proteins. Notably, we found two hydrogen bonds between the co-crystallized ligand and residues MET-111 and GLN-117, from which we speculate that these three candidate compounds have similar binding modes with the co-crystallized ligand and belong to type I inhibitors. In recent years, many researchers have conducted extensive research on inhibitors targeting JNK1 in the hope of discovering effective small-molecule inhibitors. Liu et al. utilized the HTS technique for the identification of JNK1 inhibitors and the result showed that the screened hit formed one hydrogen bond with amino acid residue MET-111 [18]. Similarly, another candidate inhibitor designed by Gong et al. interacted with residues LEU-110 and MET-111 in the hinge region through two hydrogen bonds [19]. These results are consistent with our proposed compounds.

### 2.8. Molecular Dynamics Simulation

**Figure 7:**
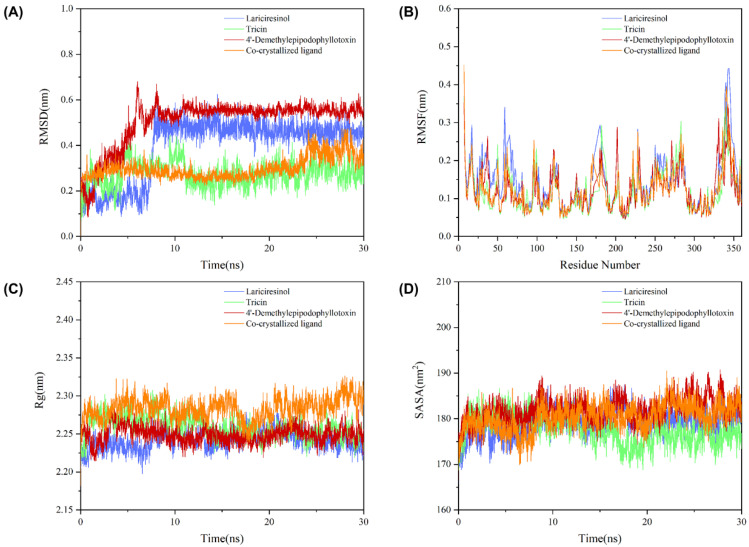
The variation in (**A**) RMSD, (**B**) RMSF, (**C**) Rg, and (**D**) SASA vs. time for each complex system during the simulation.

Molecular dynamics simulations can predict the dynamic processes of complex systems under restrictive conditions and provide a theoretical basis for the validation of experimental results. RMSD is the best parameter to detect the stability of the system as it is usually used to assess the structural shift between two conformations. As shown in Figure 7A, all systems gradually reached the equilibrium state after 10 ns. Specifically, the RMSD values of the JNK1-co-crystallized ligand and JNK1-Tricin complexes were low, while the RMSD values of the JNK1-4′-Demethylepipodophyllotoxin and JNK1-Lariciresinol complexes were generally high, which implies that the latter two systems exhibited more intense motions and greater conformational differences during the simulation. Notably, although the JNK1-4′-Demethylepipodophyllotoxin complex showed a larger degree of dynamic variation, its RMSD values fluctuated in a relatively small range, indicating that the stability of this system was slightly better. RMSF is the best parameter for the evaluation of the structural flexibility of proteins as it is often employed to measure the fluctuation of amino acid residues. As shown in Figure 7B, the trend of RMSF was roughly consistent for all systems, and the residues in region 330–350 fluctuated the most significantly. These results suggest that this region was more flexible and may be the binding site for the ligand to the protein. In addition, Rg is a common parameter to characterize the structural tightness of proteins, and SASA is an important parameter to describe the hydrophobicity of protein surfaces. As shown in Figure 7C,D, all systems displayed stable trajectories of Rg and SASA, which also explains to some extent that the protein was able to bind to the ligands stably during the simulation.

**Figure 8:**
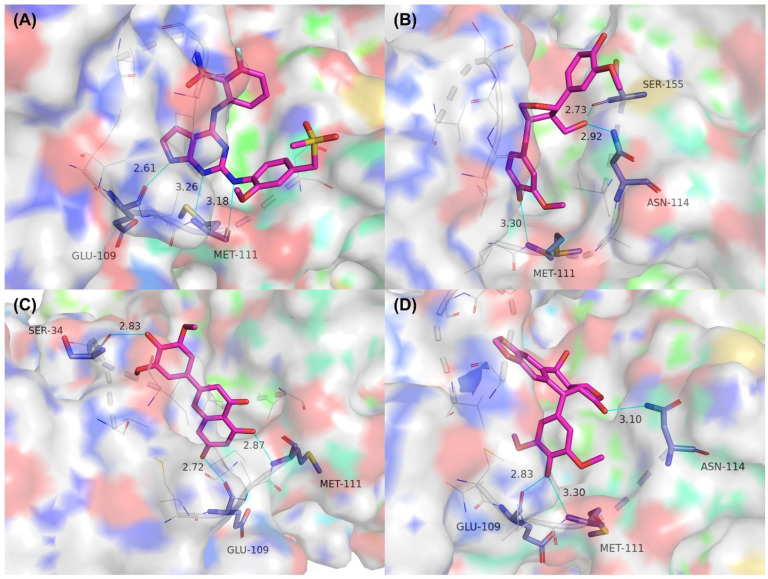
The ligand–protein interaction profiles after the simulation. (**A**) Co-crystallized ligand, (**B**) Lariciresinol, (**C**) Tricin, and (**D**) 4′-Demethylepipodophyllotoxin. The blue lines represent hydrogen bonding interactions and the gray sticks represent hydrophobic interactions.

We also analyzed the binding mode of each ligand in the active pocket of the target protein after the simulation was completed. Figure 8 depicts the results, which showed that some compounds retained the key interactions while others formed new interactions with amino acid residues. The reason for the occurrence of the above phenomenon may be the conformational changes of the complexes during the simulation. Specifically, the co-crystallized ligand lost the hydrogen bonding interaction with GLN-117 after the simulation, while the hydrogen bond with MET-111 was retained (and an additional hydrogen bond was formed). Lariciresinol retained only the interaction with MET-111. However, this compound formed two new hydrogen bonds with residues ASN-114 and SER-155 (Figure 8B). Tricin lost one hydrogen bond formed between it and MET-111 after the simulation, while other hydrogen bonds were retained (Figure 8C). 4′-Demethylepipodophyllotoxin not only retained the hydrogen bond with MET-111 but also formed new interactions with residues ASN-114 and GLU-109 (Figure 8D). It should be noted that all three candidate compounds retained hydrogen bonding interactions with the key residue MET-111 after the simulation, which may be the main reason for their stable binding.

To further investigate the binding affinity between the candidate compounds and the target protein, we calculated the binding free energy of the complexes formed by the four ligands with JNK1 using the MM/PBSA method and ranked these values based on the principle that the lower the binding free energy, the more favorable the binding. The results are as follows: co-crystallized ligand (−118.34 ± 29.11) > Lariciresinol (−84.53 ± 9.51 kJ/mol) > Tricin (−80.22 ± 10.08 kJ/mol) > 4′-Demethylepipodophyllotoxin (−78.18 ± 10.67 kJ/mol), which is consistent with molecular docking. Furthermore, the contributions of the energy terms indicate that van der Waals energy, electrostatic energy, and SASA energy play a facilitating role for drug–target binding, while polar solvation energy is detrimental to the stable binding of both (Table 3).

### 2.9. Biological Evaluation

Finally, we purchased the three candidate compounds for in vitro biological validation. The initial concentration of each compound was set at 50 µM, and the final concentration was 195 nM after a two-fold gradient dilution. The results of the kinase assay are shown in Table 4. Tricin exhibited a 75.4% inhibition of JNK1 at the concentration of 50 µM and had an IC_50_ value of 17.68 μM. In contrast, Lariciresinol and 4′-Demethylepipodophyllotoxin exhibited a relatively weak inhibition of JNK1 (IC_50_ > 50 μM). These results confirm that our virtual screening process is reliable. In addition, the proposed compound Tricin can also be used as a lead compound for the design of JNK1 inhibitors.

## 3. Discussion

JNK1 in the MAPK family is currently a key target for the treatment of type-2 diabetes. Considering the unique advantages of natural products, the identification of inhibitors targeting JNK1 from them is extremely attractive. In recent years, virtual screening methods based on computational strategies have opened new paths for the development of natural products. Machine learning algorithms have shown high success rates in the discovery of active compounds, and combining them with traditional CADD techniques could improve the efficiency as well as the reliability of virtual screening [20].

In the present study, we first constructed three machine learning classification models for the JNK1 dataset and evaluated the prediction performance of these models with different metrics. It turns out that all three models exhibit good performance on the test set, with accuracy, precision, and AUC ranging from 0.825 to 0.828, 0.748 to 0.777, and 0.891 to 0.896, respectively. In addition, exploring the spatial distribution of the dataset is an integral step. The most commonly adopted space mapping technique is PCA, which is capable of projecting data from high to low dimensions while ensuring minimal information loss [12]. The results of PCA indicated that the training set used for modeling had a wide chemical space.

As the diversity of algorithms leads to different prediction results, we integrated these three individual models based on two model fusion strategies (i.e., Voting and Stacking) to further enhance the generalization performance. The results showed that the integrated models had better performance on the test set than the individual models. Next, we used two integrated models for activity prediction of natural products in the ZINC database and performed pharmacokinetic and toxicity risk assessment. After the first round of screening, we obtained 22 potential candidate compounds.

The molecular docking-based virtual screening strategies have the obvious drawbacks of high false positive rates and slow computational speed. To overcome this problem, we narrowed down the search space using machine learning models, and, thus, only a small number of compounds needed to be docked in the second round of screening. Subsequently, a consensus analysis of the two methodologies was performed to obtain an overall score. According to the literature, we selected three compounds with known activities for further analysis. Molecular docking and molecular dynamics simulation demonstrated that these three compounds interacted with key amino acid residues and bound stably to the target protein via van der Waals and electrostatic interactions. In addition, the results of the kinase assay showed that the proposed molecule Tricin, a flavonoid isolated from *Zizania latifolia*, exhibited better inhibitory activity against JNK1 (IC_50_ = 17.68 μM).

In this study, we designed a complete virtual screening process of “machine learning-ADMET prediction-molecular docking-molecular dynamics simulation”. The program is characterized by a comprehensive combination of the advantages of traditional CADD and advanced AIDD, taking into account both the activity of the compounds and the structure of the protein, as well as analyzing the binding mode and binding stability of them. However, it is difficult to ignore that the whole process still has many limitations that need to be addressed. On the one hand, the number of compounds available for the machine learning modeling process is not sufficient. The dependence of the model on data is one of the primary constraints for the application of machine learning in the field of drug development. Considering the quality of training data sourced from various public databases has a great impact on the performance of the model, these data must be reliable to ensure the accuracy of the prediction results [21]. On the other hand, machine learning techniques are limited by the complexity of the model (the so-called “black box”), which makes it difficult to provide a reasonable interpretation or analysis of the prediction results in some cases [22]. Overall, we hope this study could provide a theoretical basis for the drug design of novel JNK1 inhibitors. In the future, we will conduct structure–activity relationship (SAR) research and further improve the activity of the screened compound through rational structural modification methods.

## 4. Materials and Methods

### 4.1. Machine Learning Dataset

#### 4.1.1. Data Preparation

We retrieved the inhibitors of JNK1 from the ChEMBL Database (https://www.ebi.ac.uk/chembl/, accessed on 27 March 2022) and the dataset was processed based on the following criteria: (i) duplicates and compounds with no identified activity were deleted; (ii) compounds with IC_50_ values less than 1 μM were marked as “active”, while the rest were marked as “inactive”. It should be noted that to ensure the reliability of the data, we only considered compounds with an assay type of “B” (i.e., directly bound to the target). After a series of preprocessing, we obtained a JNK1 dataset containing 1138 active compounds and 285 inactive compounds, which were randomly divided into training and test sets in a 4:1 ratio.

#### 4.1.2. Descriptors Pruning

Molecular descriptors mathematically characterize the chemical information of a molecule in the form of numbers [23]. With the help of the open-source toolkit RDKit (http://www.rdkit.org, accessed on 18 September 2022), we calculated 208 molecular descriptors for all compounds to determine the relationship between activity and molecular properties. Generally, in the process of machine learning model building, we need to dimensionalize the dataset (i.e., convert the data to the same specification) to avoid the impact of certain features with a particularly large range of values. Moreover, it is also crucial to select those features that contribute more to the prediction performance. Thus, we eliminated redundant and irrelevant descriptors in three steps: (i) calculate the variance of each descriptor and remove the descriptors with zero variance because these features all take the same value and are useless for sample classification; (ii) calculate the mutual information between descriptors and the IC_50_ value (label) and remove the descriptors with zero mutual information because these features are independent of the label, which not only wastes computational memory but also introduces noise [24]; (iii) calculate the Pearson correlation coefficient between all descriptors. If the value between any pair is higher than 0.8, only one of them is remained, which can avoid collinearity and enhance the interpretability of the model.

### 4.2. Machine Learning Algorithms

#### 4.2.1. Classifier Construction

Among the available machine learning algorithms, random forest (RF), support vector machine (SVM), and artificial neural network (ANN) are widely used for the discovery of lead compounds due to their excellent performance and outstanding robustness. In this study, three individual machine learning models (RF, SVM, and ANN) and two integrated machine learning models (Voting and Stacking) were constructed based on the Scikit-learn library of Python (https://scikit-learn.org/stable/, accessed on 18 September 2022). Specifically, the *RandomForestClassifier()*, *svm.SVC()*, *MLPClassifier()*, *VotingClassifier()*, and *StackingClassifier()* methods were applied.

RF, a representative ensemble algorithm based on Bagging, consists of multiple mutually independent decision trees, and the final prediction is obtained by averaging the results of overall decision trees [25]. The “randomness” of the RF algorithm is mainly reflected in two aspects: one is the formation of different training sets by random sampling with put-back (also known as bootstrapping), and the other is the use of a subset of features to split each node of the decision tree. The hyperparameters “n_estimators”, “max_depth”, and “max_features” have a substantial impact on the performance of RF.

SVM is a supervised machine learning algorithm that distinguishes between different classes of samples in a dataset by establishing a hyperplane (decision boundary) with the maximum interval [26]. The SVM algorithm provides a good balance between model complexity and learning ability, while having excellent generalization performance. The kernel function is the key to the dimensional transformation of SVM, which is divided into a linear kernel, polynomial kernel, and radial basis function (RBF). In addition, “C” and “gamma” are also significant hyperparameters of SVM.

ANN originates from the human nervous system and is composed of many interconnected nodes called artificial neurons [27]. The input signal is first processed by the nodes in the input layer and is then passed to the nodes in the hidden layer connected to it; finally, the corresponding prediction result is obtained through the nodes in the output layer. Two hyperparameters, “hidden_layer_sizes” and “alpha”, are used for the tuning of ANN.

As these machine learning algorithms have many hyperparameters, we need to obtain the best combination of them. Grid search is a traditional automatic tuning method, which searches the entire hyperparameter space exhaustively with low efficiency. The Bayesian optimization algorithm employs the Gaussian process to take full account of the prior information and perform the tuning of hyperparameters with a minimum number of iterations. In this study, we performed the tuning of the key hyperparameters mentioned above on the training set by the Bayesian optimization algorithm combined with 10-fold cross-validation.

#### 4.2.2. Classifier Evaluation

In the present study, we used five common metrics to evaluate the generated machine learning models, including accuracy, precision, recall, F1-score, and the area under the receiver operating characteristic (ROC) curve. These statistical parameters are calculated from true positives (TP), true negatives (TN), false positives (FP), and false negatives (FN). Accuracy represents the proportion of correctly predicted compounds to the total compounds. Precision represents the proportion of truly active compounds out of all compounds predicted to be active, while recall represents the proportion of the compounds that are correctly predicted by the model out of all compounds that are truly active. F1-score is the harmonic average of precision and recall. The ROC curve takes the false positive rate and recall as the horizontal and vertical coordinates (under different thresholds), respectively, and the area under the curve (AUC) is one of the most important metrics in model evaluation. They are calculated by Equations (1)–(4).

(1) $$\mathrm{Accuracy}\;=\frac{TP+TN}{TP+FP+TN+FN}$$

(2) $$\mathrm{Precision}\;=\frac{TP}{TP+FP}$$

(3) $$\mathrm{Recall}\;=\frac{TP}{TP+FN}$$

(4) $$F1-\mathrm{score}\;=\frac{2\times Precision\times Recall}{Precision+Recall}$$

### 4.3. ADMET Studies

To avoid the failure of the screened drugs at the clinical trial stage, it is necessary to perform the absorption, distribution, metabolism, elimination, and toxicity (ADMET) prediction of compounds. The online server ADMETlab 2.0 (https://admetmesh.scbdd.com/, accessed on 18 September 2022), based on the multi-task graph attention (MGA) framework for model training, is capable of calculating 88 ADMET properties, covering almost all pharmacokinetics and toxicities of interest to medicinal chemists [28]. In this study, we performed ADMET prediction for candidate compounds obtained from the machine learning models and further filtered them according to the set criterion. All drug-like molecules that met these criteria were used for molecular docking studies.

### 4.4. Molecular Docking

To calculate the binding affinity of the screened hits to JNK1, we performed a semi-flexible docking. The crystallographic structure of JNK1 was downloaded from Protein Data Bank (https://www.rcsb.org/, accessed on 18 September 2022) and preprocessed as follows: the missing atoms were inserted, the water and the co-crystallized ligand (PubChem CID: 25113171) were removed, the polar hydrogen atoms were added, and the Gasteiger charges were recalculated. The 3D structures of the small-molecule ligands were downloaded from PubChem (https://pubchem.ncbi.nlm.nih.gov/, accessed on 18 September 2022) and minimized with the MM2 force field. The grid box was generated with the co-crystallized ligand as the center, and the parameters were set as follows: center_x = 22.78, center_y = 38.34, center_z = 31.17, size_x = size_y = size_z = 12.5, and exhaustiveness = 30. To verify the feasibility of the docking process, the extracted co-crystallized ligand was re-docked into the target protein and the root-mean-square deviation (RMSD) was calculated. All candidate molecules were subjected to molecular docking studies by AutoDock Vina software [29]. After docking was completed, the protein–ligand complexes were analyzed for interactions using LigPlot^+^ software [30].

### 4.5. Consensus Analysis

To minimize the false positive rate as much as possible, we performed a consistency analysis on the results of the two rounds of screening. In this study, we defined the scores for machine learning classification models and molecular docking calculations. Specifically, the former S_(Machine Learning)_ is the average of the confidence levels of the candidate compounds in the two integrated models, and the latter S_(Molecular Docking)_ is the ratio of the binding free energies of the candidate compounds to the co-crystallized ligand, and they both take values in the range of 0–1. The final total score S_(JNK1)_ is the average of these two values.

### 4.6. Molecular Dynamics Simulation

To further investigate the dynamic stability of ligand–protein interactions, we performed molecular dynamics simulations of the complex systems using Gromacs software [31]. The topological parameters of ligands and the target protein were generated with the help of the online webserver Swiss Param (https://www.swissparam.ch/, accessed on 18 September 2022) and the pdb2gmx tool [32]. First, the protein–ligand complex was placed in the center of a cubic box with the boundary set to 1.2 nm and filled with the TIP3P water model [33]. Then, the charge of the system was balanced by adding counterions (Na^+^ and Cl^−^ ions). Next, the system was subjected to 50,000 steps of energy minimization to avoid steric collisions between the ligand and the protein. To restrict the position of the complex, the system temperature was raised to 300 K followed by the NVT equilibration, and then the system pressure was raised to 1 bar followed by the NPT equilibration. Finally, a molecular dynamics simulation was performed for 30 ns. After the simulation, the RMSD (root-mean-square deviation), RMSF (root-mean-square fluctuation), Rg (radius of gyration), and SASA (solvent accessible surface area) of each system were analyzed. Furthermore, we extracted the trajectory files from 20–30 ns stable dynamics trajectories and employed the MM/PBSA method to calculate the binding free energy of each system [34].

### 4.7. Kinase Assay

The inhibitory activity of the candidate compounds against JNK1 was measured by the Lance^®^ Ultra kinase assay from PerkinElmer. The candidate compounds were first dissolved in DMSO solution to the initial concentration, and then serially diluted two-fold for 10 points. Then, 40 µL of the compound solutions at each concentration was added to a 384-well plate. In addition, one compound-free control well and one kinase-free control well were set-up. Subsequently, 10 µL of kinase buffer solution and 10 µL of substrate solution were added to the kinase-free control well of the plate, while 10 µL of kinase solution and 10 µL of substrate solution were added to the remaining wells. The plate was incubated at room temperature for 10 min, and the reaction was stopped by adding EDTA solution. At the same time, 20 µL of the detection solution was added to the plate and incubated for 60 min. After the above procedure was completed, the Lance signal ratio (665 nm/615 nm) for each well was obtained using the Envision program, and the inhibition rate was calculated according to Equation (5). Finally, all data were imported into MS Excel and the IC_50_ values of the candidate compounds were calculated with the help of the curve fitting tool XLFit (version 5.4.0.8).

(5) $$\mathrm{Percent}\;\mathrm{inhibition}\;=\frac{\mathrm{the}\;\mathrm{Ratio}\;\mathrm{of}\;\mathrm{no}\;\mathrm{compound}\;\mathrm{control}\;-\;\mathrm{the}\;\mathrm{Ratio}\;\mathrm{of}\;\mathrm{sample}}{\mathrm{the}\;\mathrm{Ratio}\;\mathrm{of}\;\mathrm{no}\;\mathrm{compound}\;\mathrm{control}\;-\;\mathrm{the}\;\mathrm{Ratio}\;\mathrm{of}\;\mathrm{no}\;\mathrm{kinase}\;\mathrm{control}}\times 100$$

## Figures and Tables

**Table 1:** Performance metrics of the JNK1 models on training and test sets.

	Model	Accuracy	Precision	Recall	F1-Score	AUC
Training Set	RF	0.821	0.801	0.713	0.752	0.905
	SVM	0.832	0.795	0.763	0.777	0.896
	ANN	0.835	0.806	0.759	0.779	0.901
	Voting	0.836	0.811	0.752	0.778	0.913
	Stacking	0.840	0.810	0.768	0.786	0.912
Test Set	RF	0.825	0.748	0.762	0.755	0.896
	SVM	0.828	0.777	0.723	0.749	0.891
	ANN	0.825	0.763	0.733	0.748	0.891
	Voting	0.842	0.804	0.733	0.767	0.906
	Stacking	0.832	0.779	0.733	0.755	0.908

**Table 2:** The top five compounds with consensus analysis scores.

Name	CAS Number	Structure	Binding Free Energy (kcal/mol)	S_(Molecular Docking)_	Confidence Level of Voting Model	Confidence Level of Stacking Model	S_(Machine Learning)_	S_(JNK1)_
Lariciresinol	27003-73-2	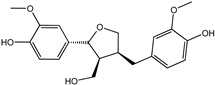	−9.282	0.873	0.855	0.861	0.858	0.866
Nigracin	18463-25-7	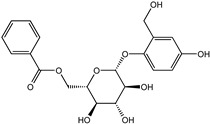	−9.229	0.868	0.829	0.838	0.834	0.851
Tricin	520-32-1	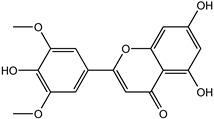	−9.007	0.847	0.830	0.836	0.833	0.840
4′-Demethylepipodophyllotoxin	6559-91-7	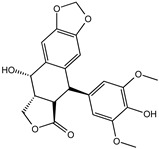	−8.856	0.833	0.826	0.859	0.842	0.838
Ophiopogonanone E	588706-66-5	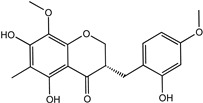	−8.802	0.828	0.844	0.846	0.845	0.837

**Table 3:** MM/PBSA binding free energy between three hits and the co-crystallized ligand with JNK1.

Compound	van der Waals Energy (kJ/mol)	Electrostatic Energy (kJ/mol)	Polar Solvation Energy (kJ/mol)	SASA Energy (kJ/mol)	Total Binding Energy (kJ/mol)
Lariciresinol	−147.82 ± 9.72	−39.20 ± 7.35	119.25 ± 8.46	−16.74 ± 0.82	−84.53 ± 9.51
Tricin	−142.25 ± 10.38	−18.34 ± 7.86	97.03 ± 12.46	−16.55 ± 1.00	−80.22 ± 10.08
4′-Demethylepipodophyllotoxin	−127.96 ± 9.74	−34.25 ± 8.06	98.59 ± 14.58	−14.48 ± 0.84	−78.18 ± 10.67
Co-crystallized ligand	−172.17 ± 18.60	−98.91 ± 30.84	169.53 ± 18.81	−16.80 ± 1.30	−118.34 ± 29.11

**Table 4:** The inhibition rates of candidate compounds against JNK1 at the first five concentration points.

Compound	50 μM	25 μM	12.5 μM	6.25 μM	3.125 μM	IC_50_
Lariciresinol	15.1%	11.6%	9.0%	7.2%	5.6%	>50 μM
Tricin	75.4%	61.8%	47.2%	34.8%	25.9%	17.68 μM
4′-Demethylepipodophyllotoxin	10.2%	7.9%	5.7%	3.3%	1.9%	>50 μM

## Data Availability

The data that support the findings of this study are available from the corresponding author upon reasonable request.

## Supplementary Materials

The following supporting information can be downloaded at: https://www.mdpi.com/article/10.3390/molecules27196249/s1, Figure S1: Relative importance ranking of the 10 molecular descriptors most associated with JNK1 inhibitory activity; Figure S2: Chemical spatial distribution of the trained compounds and predicted compounds; Table S1: The details of the descriptors after feature selection.

## Sample Availability

Samples of the compounds Lariciresinol, Tricin, and 4′-Demethylepipodophyllotoxin are available from the authors.
