# Supplementary material for: Discovery of Novel c-Jun N-Terminal Kinase 1 Inhibitors from Natural Products: Integrating Artificial Intelligence with Structure-Based Virtual Screening and Biological Evaluation
Source: Molecules. 2022 Sep 22;27(19):6249. doi: 10.3390/molecules27196249 (PMC9572546; doi:10.3390/molecules27196249)
Supplement: Supplementary file 1 [file molecules-27-06249-s001.zip › molecules-1914335-supplementary.pdf]

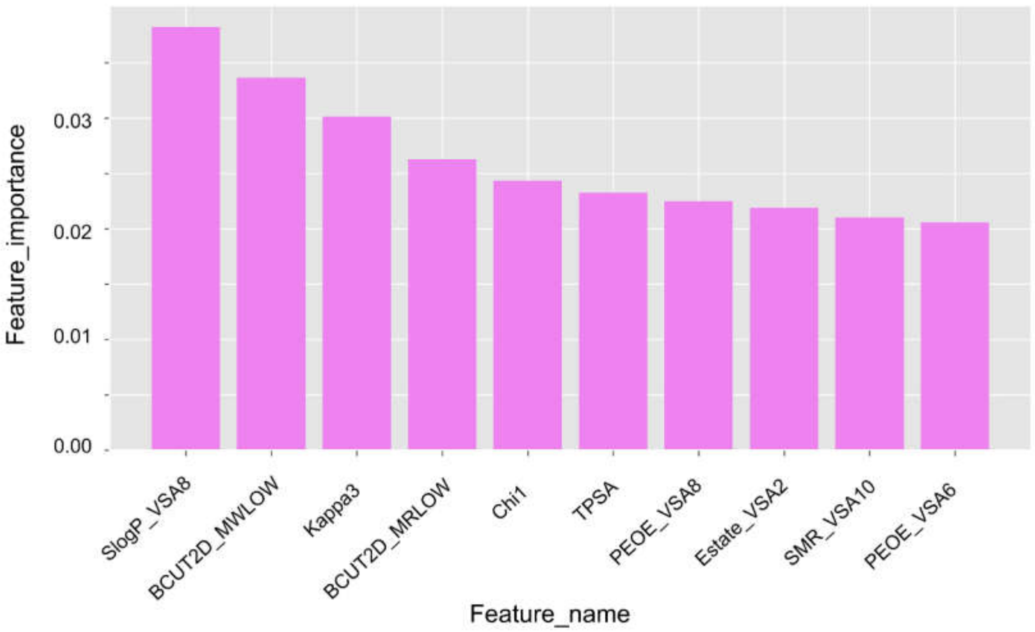

Figure S1. Relative importance ranking of the 10 molecular descriptors most associated with JNK1 inhibitory activity.

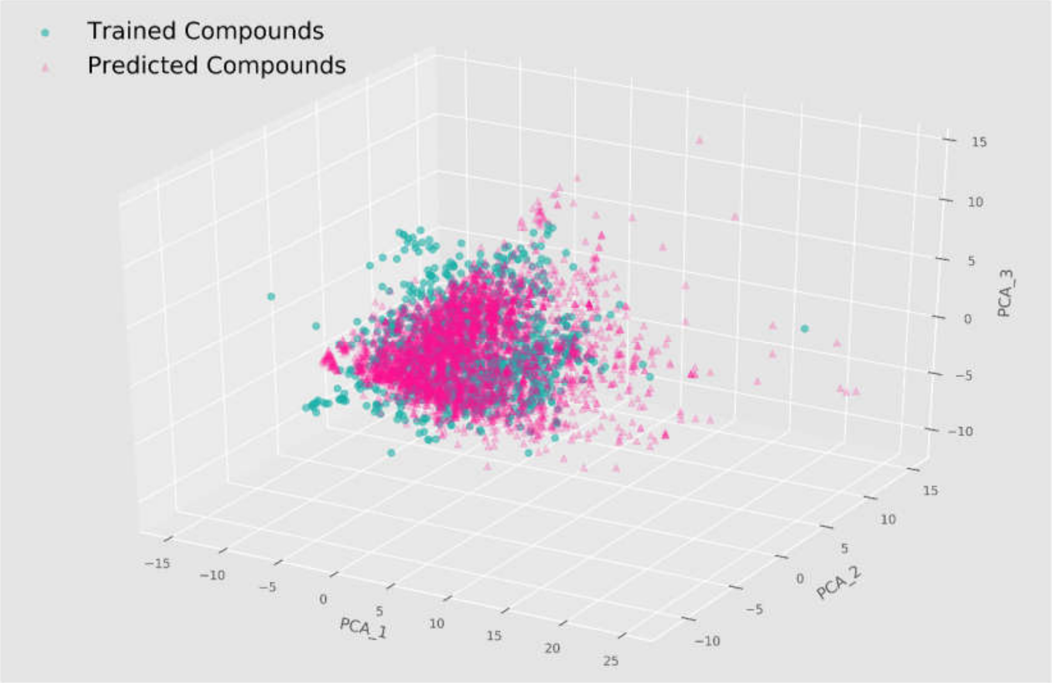

Figure S2. Chemical spatial distribution of the trained compounds and predicted compounds.

Table S1. The details of the descriptors after feature selection.

Descriptor Name	Type	Dimention	Extended class
BalabanJ	Balaban's J index	2	Topological descriptors
Chi1	Chi indices	2	Connectivity descriptors
Chi2v	Chi indices	2	Connectivity descriptors
Chi4n	Chi indices	2	Connectivity descriptors
EState_VSA1	EState_VSA	2	MOE-type descriptors
EState_VSA2	EState_VSA	2	MOE-type descriptors
EState_VSA3	EState_VSA	2	MOE-type descriptors
EState_VSA4	EState_VSA	2	MOE-type descriptors
EState_VSA5	EState_VSA	2	MOE-type descriptors
EState_VSA6	EState_VSA	2	MOE-type descriptors
EState_VSA7	EState_VSA	2	MOE-type descriptors
EState_VSA8	EState_VSA	2	MOE-type descriptors
EState_VSA9	EState_VSA	2	MOE-type descriptors
EState_VSA10	EState_VSA	2	MOE-type descriptors
FractionCSP3	FractionCSP3	1	Constitutional descriptors
HallKierAlpha	HallKierAlpha	2	Topological descriptors
Ipc	Ipc	2	Topological descriptors
Kappa3	Kappa descriptors	2	Topological descriptors
LabuteASA	LabuteASA	2	MOE-type descriptors
MolLogP	MolLogP	2	Molecular property descriptors
NHOHCount	NHOHCount	1	Constitutional descriptors
NOCount	NOCount	1	Constitutional descriptors
NumAliphaticCarbocycles	NumAliphaticCarbocycles	1	Constitutional descriptors
NumAliphaticHeterocycles	NumAliphaticHeterocycles	1	Constitutional descriptors
NumAromaticHeterocycles	NumAromaticHeterocycles	1	Constitutional descriptors
NumAromaticRings	NumAromaticRings	1	Constitutional descriptors
NumHAcceptors	NumHAcceptors	1	Constitutional descriptors
NumHeteroatoms	NumHeteroatoms	1	Constitutional descriptors
NumRotatableBonds	NumRotatableBonds	1	Constitutional descriptors
NumSaturatedRings	NumSaturatedRings	1	Constitutional descriptors
BCUT2D_MWHI	BCUT	2	BCUT descriptors
BCUT2D_MWLOW	BCUT	2	BCUT descriptors
BCUT2D_CHGHI	BCUT	2	BCUT descriptors
BCUT2D_CHGLO	BCUT	2	BCUT descriptors
BCUT2D_LOGPHI	BCUT	2	BCUT descriptors
BCUT2D_LOGPLOW	BCUT	2	BCUT descriptors
BCUT2D_MRHI	BCUT	2	BCUT descriptors
BCUT2D_MRLOW	BCUT	2	BCUT descriptors
PEOE_VSA1	PEOE_VSA	2	MOE-type descriptors

PEOE_VSA2	PEOE_VSA	2	MOE-type descriptors
PEOE_VSA3	PEOE_VSA	2	MOE-type descriptors
PEOE_VSA4	PEOE_VSA	2	MOE-type descriptors
PEOE_VSA5	PEOE_VSA	2	MOE-type descriptors
PEOE_VSA6	PEOE_VSA	2	MOE-type descriptors
PEOE_VSA7	PEOE_VSA	2	MOE-type descriptors
PEOE_VSA8	PEOE_VSA	2	MOE-type descriptors
PEOE_VSA9	PEOE_VSA	2	MOE-type descriptors
PEOE_VSA10	PEOE_VSA	2	MOE-type descriptors
PEOE_VSA11	PEOE_VSA	2	MOE-type descriptors
PEOE_VSA13	PEOE_VSA	2	MOE-type descriptors
RingCount	RingCount	1	Constitutional descriptors
SMR_VSA1	SMR_VSA	2	MOE-type descriptors
SMR_VSA2	SMR_VSA	2	MOE-type descriptors
SMR_VSA3	SMR_VSA	2	MOE-type descriptors
SMR_VSA4	SMR_VSA	2	MOE-type descriptors
SMR_VSA5	SMR_VSA	2	MOE-type descriptors
SMR_VSA6	SMR_VSA	2	MOE-type descriptors
SMR_VSA7	SMR_VSA	2	MOE-type descriptors
SMR_VSA9	SMR_VSA	2	MOE-type descriptors
SMR_VSA10	SMR_VSA	2	MOE-type descriptors
SlogP_VSA1	SlogP_VSA	2	MOE-type descriptors
SlogP_VSA2	SlogP_VSA	2	MOE-type descriptors
SlogP_VSA3	SlogP_VSA	2	MOE-type descriptors
SlogP_VSA4	SlogP_VSA	2	MOE-type descriptors
SlogP_VSA5	SlogP_VSA	2	MOE-type descriptors
SlogP_VSA8	SlogP_VSA	2	MOE-type descriptors
SlogP_VSA10	SlogP_VSA	2	MOE-type descriptors
SlogP_VSA11	SlogP_VSA	2	MOE-type descriptors
SlogP_VSA12	SlogP_VSA	2	MOE-type descriptors
TPSA	TPSA	2	Molecular property descriptors
VSA_EState1	VSA_Estate	2	MOE-type descriptors
VSA_EState2	VSA_Estate	2	MOE-type descriptors
VSA_EState3	VSA_Estate	2	MOE-type descriptors
VSA_EState4	VSA_Estate	2	MOE-type descriptors
VSA_EState5	VSA_Estate	2	MOE-type descriptors
VSA_EState6	VSA_Estate	2	MOE-type descriptors
VSA_EState7	VSA_Estate	2	MOE-type descriptors
VSA_EState8	VSA_Estate	2	MOE-type descriptors
VSA_EState9	VSA_Estate	2	MOE-type descriptors
VSA_EState10	VSA_Estate	2	MOE-type descriptors
fr_Al_COO	fr_Al_COO	1	Constitutional descriptors
fr_Al_OH	fr_Al_OH	1	Constitutional descriptors
fr_Ar_N	fr_Ar_N	1	Constitutional descriptors

fr_Ar_OH	fr_Ar_OH	1	Constitutional descriptors
fr_COO	fr_COO	1	Constitutional descriptors
fr_C_O	fr_C_O	1	Constitutional descriptors
fr_C_S	fr_C_S	1	Constitutional descriptors
fr_NH0	fr_NH0	1	Constitutional descriptors
fr_NH1	fr_NH1	1	Constitutional descriptors
fr_Ndealkylation2	fr_Ndealkylation2	1	Constitutional descriptors
fr_Nhpyrrole	fr_Nhpyrrole	1	Constitutional descriptors
fr_SH	fr_SH	1	Constitutional descriptors
fr_allylic_oxid	fr_allylic_oxid	1	Constitutional descriptors
fr_amide	fr_amide	1	Constitutional descriptors
fr_amidine	fr_amidine	1	Constitutional descriptors
fr_aniline	fr_aniline	1	Constitutional descriptors
fr_aryl_methyl	fr_aryl_methyl	1	Constitutional descriptors
fr_azo	fr_azo	1	Constitutional descriptors
fr_diazo	fr_diazo	1	Constitutional descriptors
fr_benzene	fr_benzene	1	Constitutional descriptors
fr_ester	fr_ester	1	Constitutional descriptors
fr_ether	fr_ether	1	Constitutional descriptors
fr_furan	fr_furan	1	Constitutional descriptors
fr_halogen	fr_halogen	1	Constitutional descriptors
fr_ketone_Topliss	fr_ketone_Topliss	1	Constitutional descriptors
fr_morpholine	fr_morpholine	1	Constitutional descriptors
fr_nitro	fr_nitro	1	Constitutional descriptors
fr_oxazole	fr_oxazole	1	Constitutional descriptors
fr_para_hydroxylation	fr_para_hydroxylation	1	Constitutional descriptors
fr_priamide	fr_priamide	1	Constitutional descriptors
fr_pyridine	fr_pyridine	1	Constitutional descriptors
fr_sulfonamd	fr_sulfonamd	1	Constitutional descriptors
fr_sulfone	fr_sulfone	1	Constitutional descriptors
fr_tetrazole	fr_tetrazole	1	Constitutional descriptors
fr_urea	fr_urea	1	Constitutional descriptors
MaxEStateIndex	Estate Index	2	Topological descriptors
MaxPartialCharge	Partial Charge	2	Topological descriptors
MinAbsEStateIndex	Estate Index	2	Topological descriptors
MinEStateIndex	Estate Index	2	Topological descriptors
MinPartialCharge	Partial Charge	2	Topological descriptors
